# Inhibitory and excitatory axon terminals share a common nano-architecture of their Ca_v_2.1 (P/Q-type) Ca^2+^ channels

**DOI:** 10.3389/fncel.2015.00315

**Published:** 2015-08-11

**Authors:** Daniel Althof, David Baehrens, Masahiko Watanabe, Noboru Suzuki, Bernd Fakler, Ákos Kulik

**Affiliations:** ^1^Institute of Physiology, University of FreiburgFreiburg, Germany; ^2^Department of Anatomy, Graduate School of Medicine, Hokkaido UniversitySapporo, Japan; ^3^Department of Animal Genomics, Functional Genomics Institute, Mie UniversityMie, Japan; ^4^Centre for Biological Signalling Studies, University of FreiburgFreiburg, Germany

**Keywords:** Ca^2+^ channels, quantitative immunoelectron microscopy, cluster analysis, rat, hippocampus

## Abstract

Tuning of the time course and strength of inhibitory and excitatory neurotransmitter release is fundamental for the precise operation of cortical network activity and is controlled by Ca^2+^ influx into presynaptic terminals through the high voltage-activated P/Q-type Ca^2+^ (Ca_v_2.1) channels. Proper channel-mediated Ca^2+^-signaling critically depends on the topographical arrangement of the channels in the presynaptic membrane. Here, we used high-resolution SDS-digested freeze-fracture replica immunoelectron microscopy together with automatized computational analysis of Ca_v_2.1 immunogold labeling to determine the precise subcellular organization of Ca_v_2.1 channels in both inhibitory and excitatory terminals. Immunoparticles labeling the pore-forming α1 subunit of Ca_v_2.1 channels were enriched over the active zone of the boutons with the number of channels (3–62) correlated with the area of the synaptic membrane. Detailed analysis showed that Ca_v_2.1 channels are non-uniformly distributed over the presynaptic membrane specialization where they are arranged in clusters of an average five channels per cluster covering a mean area with a diameter of about 70 nm. Importantly, clustered arrangement and cluster properties did not show any significant difference between GABAergic and glutamatergic terminals. Our data demonstrate a common nano-architecture of Ca_v_2.1 channels in inhibitory and excitatory boutons in stratum radiatum of the hippocampal CA1 area suggesting that the cluster arrangement is crucial for the precise release of transmitters from the axonal boutons.

## Introduction

A balance between inhibitory and excitatory synaptic transmission is essential for the normal functioning of cortical neuronal circuits. The net effect of synaptic inhibition and excitation is determined by the firing properties of inhibitory GABAergic and excitatory glutamatergic cells as well as by the release dynamics of GABA- and glutamate-filled vesicles. The transmitter release is primarily triggered by Ca^2+^ influx through voltage-gated Ca^2+^ (Ca_v_) channels (Clapham, 2007) that are activated by action potentials and/or sub-threshold depolarizing signals (Schneggenburger and Neher, 2005; Nadkarni et al., 2010). Consequently, number, density and spatial relationship of Ca_v_ channels relative to the active zone of the presynaptic boutons, the actual locus of vesicle fusion, are assumed to be crucial factors in fine-tuning the temporal precision of transmitter release (Eggermann et al., 2012; Scimemi and Diamond, 2012; Sheng et al., 2012). At fast mammalian central synapses the subfamily two Ca_v_ channels, Ca_v_2.1 (P/Q-type) and Ca_v_2.2 (N-type), are essential for coupling the presynaptic action potential to transmitter release (Wu and Saggau, 1994; Stevens, 2004; Cao and Tsien, 2010; Ariel et al., 2013) thus controlling the efficacy of transmission (Poncer et al., 1997; Catterall and Few, 2008; Lipscombe et al., 2013).

In the CA1 area of the hippocampus, pyramidal cells are under the control of inhibitory GABAergic and excitatory glutamatergic cells. GABAergic inputs originating mainly from local interneurons, controlling the firing rate of pyramidal cells and modulate their spike timing as well as synchronize their activity (Klausberger, 2009). In contrast, glutamatergic inputs arriving predominantly from pyramidal cells in CA3 and entorhinal cortex carry predictions based on memory recall and sensory information, respectively (Lisman, 1999; Otmakhova and Lisman, 2004). Thus, inhibitory and excitatory projections, targeting different subcellular domains of the CA1 principal cells, exert distinct effects on concerted and synchronous activities of hippocampal neurons and overall on rhythmic brain activities by released GABA and glutamate. The amount and kinetics of neurotransmitter release related to the intracellular Ca^2+^ concentration ([Ca^2+^]_i_) needs to be tightly regulated in terminals by Ca^2+^ entry through Ca_v_ channels, as even small changes in presynaptic Ca^2+^ influx lead to large changes in vesicle release and neurotransmission (Frank, 2014). This raises the question of whether hippocampal inhibitory and excitatory synapses are similar or fundamentally different regarding the subcellular organization of the Ca_v_ channels. The Ca^2+^-dependent synchronous release of neurotransmitters require the concerted compliance of various functionally interacting proteins forming the Ca_v_2 channel-associated networks, termed nano-environment (Müller et al., 2010), in presynaptic compartments. The spatial arrangements of these specific proteins may determine the two-dimensional distribution pattern of Ca_v_2 channels in membrane segments of axonal boutons thereby placing the channel at a position optimal for triggering the release machinery. Although, recent functional studies achieved substantial progress in localizing Ca_v_2 channels in cortical inhibitory (Bucurenciu et al., 2008; Kisfali et al., 2013) and excitatory (Kulik et al., 2004; Holderith et al., 2012; Parajuli et al., 2012; Indriati et al., 2013; Baur et al., 2015) synapses as well as at the calyx of Held (Nakamura et al., 2015), qualitative and quantitative comparison of Ca_v_2.1 channel topographical arrangement in small presynaptic boutons in the CA1 area of the hippocampus remained unresolved.

Here, we combined the high-resolution sodium dodecyl sulfate-digested freeze-fracture replica labeling (SDS-FRL) immunoelectron microscopy with automatized computational cluster analysis of immunoreactivity to determine the number and the spatial distribution profile of Ca_v_2.1 channels in terminals of both GABAergic and glutamatergic cells in the stratum radiatum of the hippocampal CA1 region.

## Materials and Methods

### Sodium Dodecyl Sulfate-Digested Freeze-Fracture Replica Immunolabeling (SDS-FRL) and Electron Microscopy

#### Immunolabeling

For the current study 6-week-old male Wistar rats (*n* = 6), one adult male Ca_v_2.1 knock-out (ko) mouse, and one adult male wild type (wt) mouse were used. The perfusion of the animals and preparation of tissues and replicas for SDS-FRL were performed as described previously (Kulik et al., 2006; Masugi-Tokita and Shigemoto, 2007). Care and handling of the animals prior to and during the experimental procedures followed European Union regulations and was approved by the Animal Care and Use Committees of the authors’ institutions. Animals were anesthetized with sodium pentobarbital (50 mg/kg, i.p.), and the hearts were surgically exposed for perfusion fixation. First, the vascular system was flushed by 25 mM phosphate-buffered saline (PBS) followed by transcardial perfusion with a fixative containing 2% paraformaldehyde (Merck, Germany) and 15% saturated picric acid made up in 0.1 M phosphate buffer (PB). Sagittal sections from the CA1 area were cut on a microslicer at a thickness of 110 μm. The slices were cryoprotected in a solution containing 30% glycerol made up in 0.1 M PB and then frozen by a high-pressure freezing machine (HPM 100, Leica, Austria). Frozen samples were inserted into a double replica table and then fractured into two pieces at -130°C. Fractured faces were replicated by deposition of carbon (5 nm thickness), platinum (2 nm), and carbon (18 nm) in a freeze-fracture replica machine (BAF 060, BAL-TEC, Lichtenstein). They were digested in a solution containing 2.5% SDS and 20% sucrose made up in 15 mM Tris buffer (TB), pH 8.3, at 80°C for 18 h. Replicas were washed in 50 mM Tris-buffered saline (TBS) containing 0.05% BSA (Roth, Germany) and 0.1% Tween20 (Tw20, Roth) and then incubated in a blocking solution (5% BSA) and then in mixtures of primary antibodies: (i) Ca_v_2.1 [Guinea pig (Gp), 5 μg/ml] and RIM1/2 [Rabbit (Rb), 1 μg/ml; Synaptic System, Göttingen, Germany], (ii) vesicular GABA transporter (VGAT, Gp, 4.5 μg/ml) and Ca_v_2.1 (Rb, 1 μg/ml; Synaptic System, Göttingen), (iii) vesicular glutamate transporter-1 (VGLUT-1, Rb, 6 μg/ml) and Ca_v_2.1 (Gp, 5 μg/ml), (iv) VGAT (Gp, 4.5 μg/ml) and VGLUT-1 (Rb, 6 μg/ml) in 50 mM TBS containing 1% BSA and 0.1% Tw20 overnight (O/N) at room temperature. Replicas were reacted with a mixture of gold-coupled (10 and 15 nm or 5 and 10 nm) goat anti-guinea pig and goat anti-rabbit IgGs secondary antibodies (1:30; BioCell Research Laboratories, Cardiff, UK) made up in 50 mM TBS containing 5% BSA O/N at 15°C.

#### Electron Microscopy

The labeled replicas were examined using a transmission electron microscope (Philips CM100).

#### Control Experiments

The specificity of immunolabeling for Ca_v_2.1 was controlled by staining of sections obtained from wt and ko mice. In wt animals [VGAT-Ca_v_2.1 (*n* = 76 terminals; VGLUT-1-Ca_v_2.1 (*n* = 40)] the pattern of immunostaining was identical to that of rat, whereas in ko mouse [VGAT-Ca_v_2.1 (*n* = 68); VGLUT-1-Ca_v_2.1 (*n* = 53)] no immunolabeling for the channel subunit was detected further confirming the specificity of the antibodies.

### Quantification of Immunogold Distribution

The distribution of immunogold labeling for Ca_v_2.1 was evaluated using an in-house developed automatized computational procedure. As an input, the underlying algorithm used x- and y-coordinates (in pixels) of the particles that were extracted from electron micrographs with the ImageJ software package (Schneider et al., 2012). The plasma membrane area covered with immunoparticles was calculated using the convex hull, the smallest area containing all particles as well as every line segment between all pairs of particles that was determined with the QuickHull algorithm (Barber et al., 1996). The cluster-assignment was obtained from the single-link method (Sibson, 1973). Accordingly, particles are assigned to the same cluster if their distances fall below a given threshold length that was set to 21 nm around the center of a gold particle. This distance is equivalent to the combined length of the radius of the 10 nm gold particle and the lengths of primary and secondary antibodies (2 × 8 nm = length of two IgGs, **Figure 4A**) (Amiry-Moghaddam and Ottersen, 2013).

To validate the clustering of Ca_v_2.1 immunoparticles our computational procedure was applied to random samples that were generated as follows: for each putative active zone, an equal number of control particles was randomly placed within a frame defined by the coordinates of the outer most particles (**Figure 4D**). When the size of the putative active zone was large enough while maintaining an equal number of particles they were placed not closer than 10 nm, which corresponds to the diameter of a gold particle and avoids overlap of two neighboring particles. The final random control for any active zone was the average of ten individually generated random distributions.

#### Control Experiments

To assure that clustering of Ca_v_2.1 channels is not due to an artifact by the secondary antibody we investigated the subcellular distribution of VGAT and VGLUT-1 as well as the GluRδ2 receptor by using the same 10 nm gold-coupled secondary antibodies. These proteins showed a distribution pattern different from Ca_v_2.1 and did not form clusters. The automatized computational cluster analysis of immunoreactivity for GluRδ2 showed no significant difference compared to a random uniform sampling regarding both cohesion (*p* = 0.069) and separation (*p* = 0.86).

### Statistical Analysis

Immunoreactivity for Ca_v_2.1 was quantitatively analyzed in putative active zones of GABAergic, putative glutamatergic and glutamatergic boutons (*n* = 54 for VGAT+, *n* = 67 for VGAT-, *n* = 90 for VGLUT-1+) obtained from two animals. Absolute numbers of Ca_v_2.1 immunoparticles per active zone were compared using the Mann–Whitney test. Correlation of the number of Ca_v_2.1 and the respective convex hull area was determined by the Spearman coefficient of correlation (r_s_). Clusters of immunogold particles in VGAT+ and VGAT- boutons as well as in VGAT+ and VGLUT-1+ boutons as regards cohesion and separation were compared using the cumulative probability distributions and by performing the two-sample Kolmogorov–Smirnov test. The number of clusters per active zone, particles per clusters, and diameter of clusters in VGAT+ and VGAT- boutons as well as in VGAT+ and VGLUT-1+ boutons were statistically compared using the two-sample Kolmogorov–Smirnov test. Statistical significance was assessed by a *p*-value threshold of 0.05.

## Results

### Ca_v_2.1 Protein is Localized to the Active Zones of Axon Terminals

**FIGURE 1:**
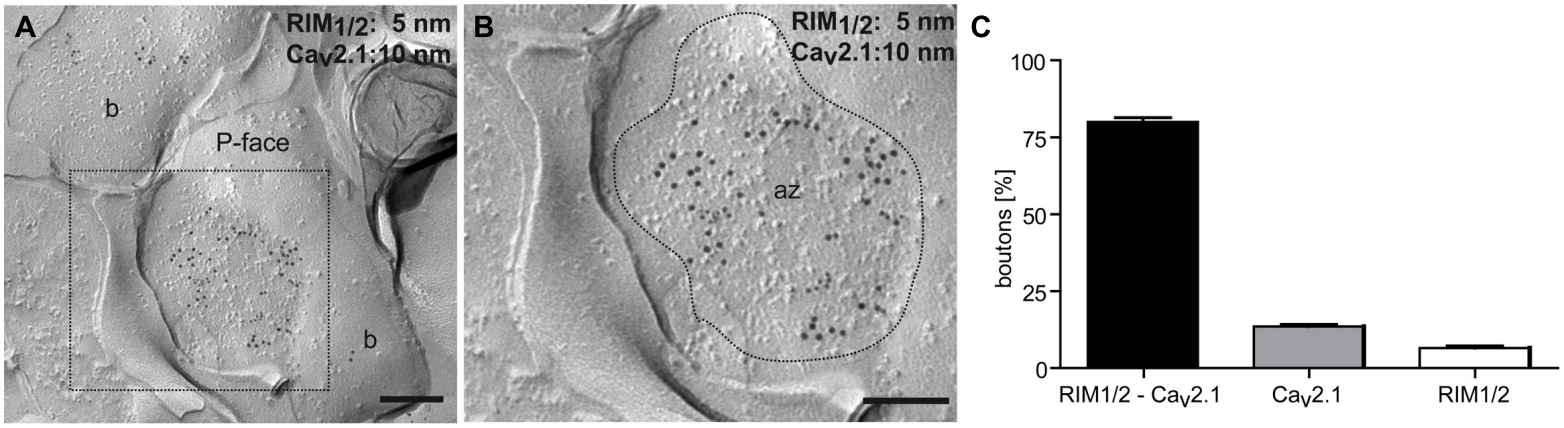
**Ca_v_2.1 localized to the active zone of axon terminals of hippocampal cells. (A,B)** Colocalization of Ca_v_2.1 (10 nm gold particles) and the presynaptic marker proteins RIM1/2 (5 nm gold particles) in the active zone (az) of a bouton (b; inset, **B**) as assessed by the SDS-FRL method. The presynaptic active zone is indicated by the high density of intramembrane particles (IMPs) on the concave shape of the protoplasmic face (P-face) of the membrane (delineated by broken line). **(C)** Bar graph summarizing labeling and co-labeling of Ca_v_2.1 and RIM1/2 in 303 axon terminals. Note co-labeling in the majority of terminals (81%). Scale bars, 0.2 μm.

All antibodies used target intracellular epitopes and, therefore, result in labeling of the protoplasmic face (P-face) of the replicas. First, we determined the distribution of the Ca_v_2.1 channels at presynaptic sites. Immunogold labeling for the channel’s pore-forming α1 subunit was observed in the active zone of axon terminals that were recognized by their high density of intramembrane particles (IMPs) on the P-face of the invaginated plasma membrane and were identified by immunolabeling for the presynaptic marker proteins RIM1/2 (**Figures 1A,B**). Quantitative analysis further revealed a high degree of co-localization of Ca_v_2.1 and RIM1/2 in the majority (81%) of the investigated terminals (*n* = 303; **Figure 1C**), indicating that Ca_v_2.1 channels are mainly confined to the active zone of boutons in the stratum radiatum of CA1.

### Inhibitory and Excitatory Boutons Show Similar Arrangement of Ca_v_2.1

**FIGURE 2:**
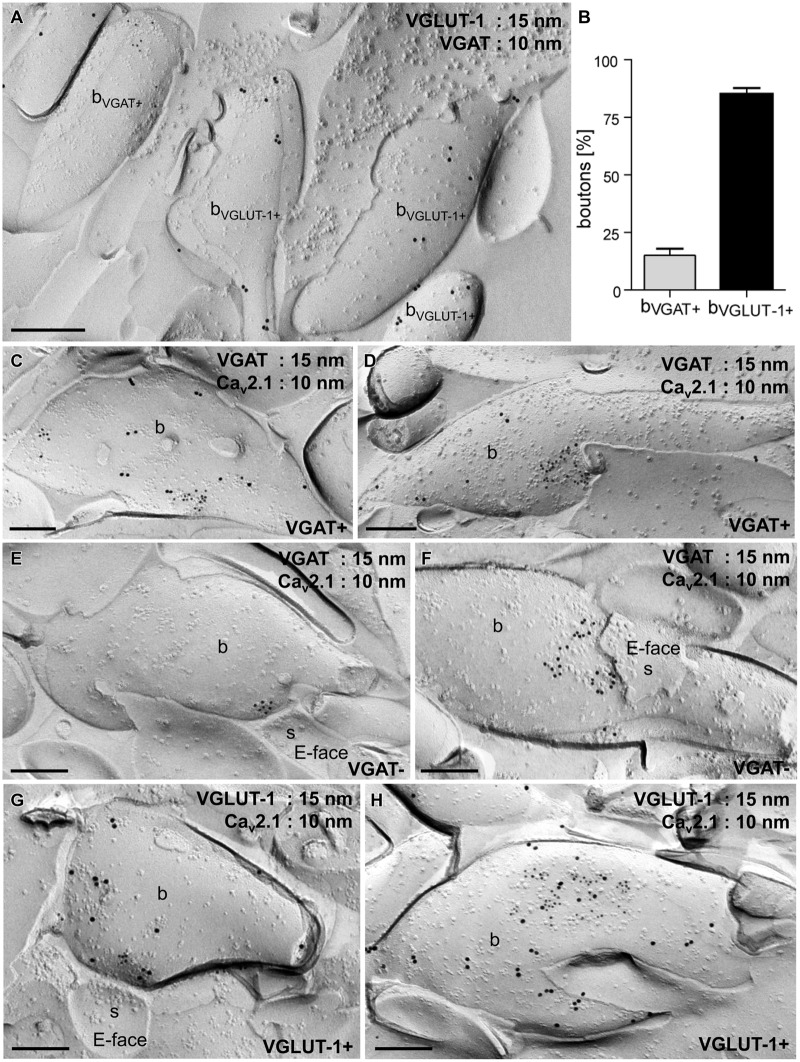
**Ca_v_2.1 channels are organized in discrete groups in the presynaptic active zone of boutons in inhibitory GABAergic and excitatory glutamatergic cells in the stratum radiatum of the hippocampal CA1 area. (A)** Electron micrograph of a replica double-labeled for vesicular GABA transporter (VGAT; 10 nm gold particles) and VGLUT-1 (15 nm) showing no overlap between the two subpopulations. **(B)** Quantification of gold particles further demonstrated that 15% of the examined axon terminals (*n* = 328) were VGAT+ (b_VGAT+_) and 85% were immunoreactive for VGLUT-1 (b_VGLUT-1+_). **(C–H)** Replica images showing aggregation of immunogold particles labeling Ca_v_2.1 (10 nm) in small **(C,E,G)** and large **(D,F,H)** active zones of VGAT+ (15 nm; **C,D**), VGAT- **(E,F)** and VGLUT-1+ (15 nm; **G,H**) boutons (b). Note that VGAT- and VGLUT-1+ terminals make asmmetrical synapses with dendritic spines (s in **E,F,G**) that can be recognized by the high density of IMPs on the E-face of the plasma membrane. Scale bars, 0.2 μm.

Next we compared the distribution of immunoparticles labeling Ca_v_2.1 in axon terminals of inhibitory GABAergic and excitatory glutamatergic neurons. For this purpose, three series of double immunolabeling experiments were performed: (i) labeling for vesicular GABA transporter (VGAT) and vesicular glutamate transporter-1 (VGLUT-1), (ii) labeling for VGAT and Ca_v_2.1, and (iii) labeling for VGLUT-1 and Ca_v_2.1. Immunoreactivity for VGAT and VGLUT-1 appeared in two non-overlapping subpopulations of boutons (**Figure 2A**): 15% of the terminals (*n* = 328) showed immunoreactivity for VGAT, while 85% of them were labeled for VGLUT-1 (**Figure 2B**). To directly compare the localization of Ca_v_2.1 in GABAergic and glutamatergic terminals we then analyzed replicas double labeled for VGAT and the channel subunit. Inhibitory terminals were recognized from immunoreactivity for VGAT (VGAT+; **Figures 2C,D**), whereas VGAT- putative excitatory boutons were adjacent to postsynaptic dendritic spines that were characterized by a high density of IMPs on the exoplasmic face (E-face) of the membrane (**Figures 2E,F**) that represent AMPA-type glutamate receptors in the postsynaptic membrane of asymmetrical synapses (Holderith et al., 2012). Immunoparticles for Ca_v_2.1 were highly concentrated in the synaptic membrane and were distributed non-homogeneously over small (**Figures 2C,E**) and large (**Figures 2D,F**) active zones of the terminals of both populations of neurons. Similar to VGAT- terminals, excitatory boutons, visualized by immunoreactivity for VGLUT-1 (VGLUT-1+), showed a non-homogeneous pattern for Ca_v_2.1 distribution: gold particles labeling the channel protein were confined to the presynaptic membrane specialization where they formed discrete groups throughout the active zones (**Figures 2G,H**).

These results indicate that Ca_v_2.1 channels display similar distribution patterns with clustered appearance in the synaptic membrane of axon terminals of both GABAergic and glutamatergic neurons in the stratum radiatum of CA1.

**FIGURE 3:**
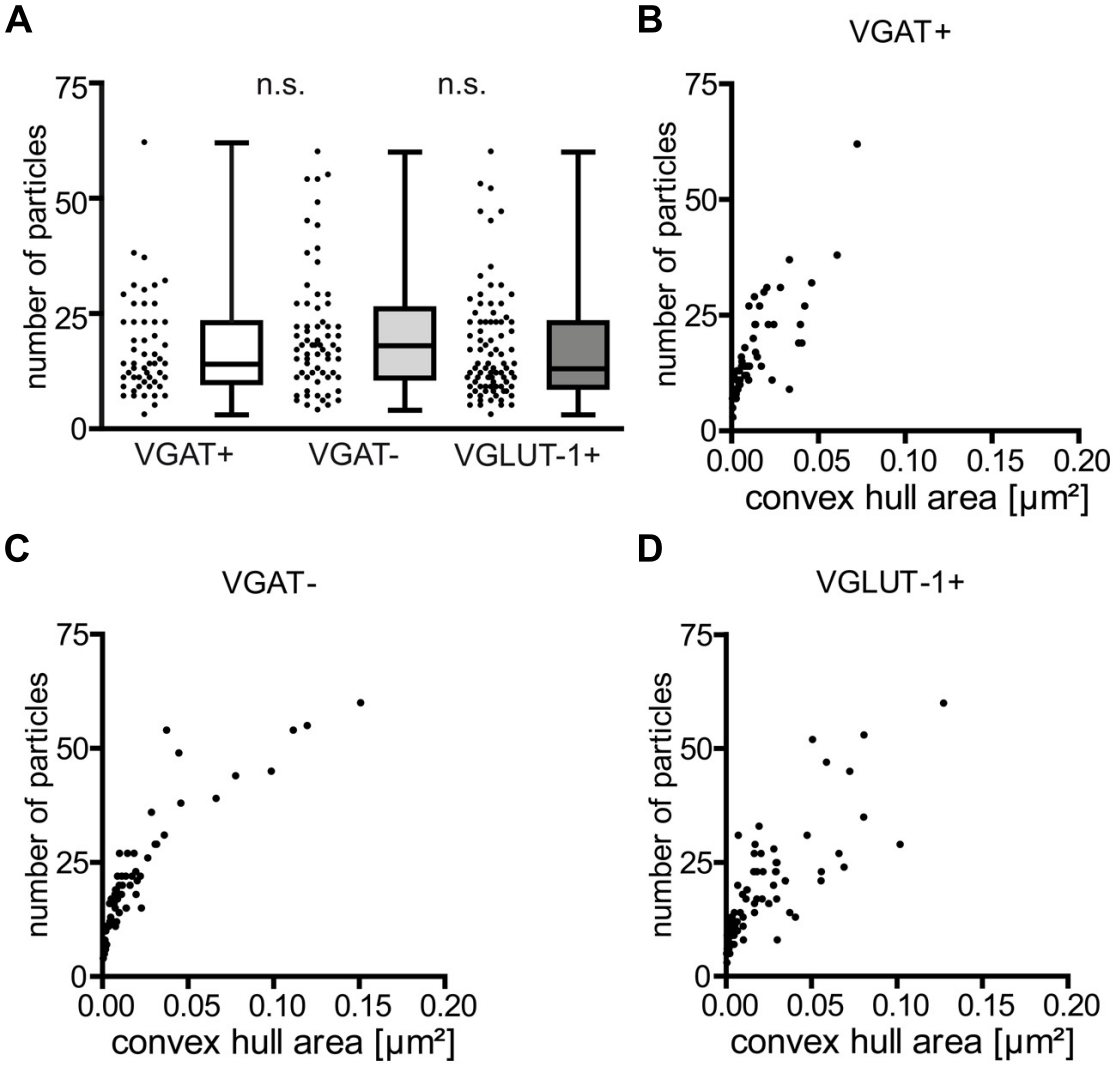
**The number of immunoparticles for Ca_v_2.1 channels is highly variable and proportional to the active zone area of the boutons. (A)** Summary plot (scatter plot: single values, box-, and whisker plots: median, interquartile range (iqr) as well as minimum and maximum) of Ca_v_2.1 particles in the indicated terminals. Note the lack of differences between the distinct types of boutons (*p* = 0.22 and *p* = 0.11), Mann–Whitney test between VGAT+ and VGAT- and between VGAT- and VGLUT-1+ terminals. **(B–D)** The number of immunogold particles labeling Ca_v_2.1 strongly correlated with the convex hull area of both GABAergic and glutamatergic boutons [Spearman correlation coefficient (*r*_s_) = 0.83 for VGAT+; *r*_s_ = 0.92 for VGAT-; *r*_s_ = 0.86 for VGLUT-1+].

Next we determined the absolute number of Ca_v_2.1 immunogold particles in the presynaptic active zones and correlated them with the convex hull area of either type of bouton. These analyses showed that the number of Ca_v_2.1 immunogold particles was highly variable ranging from 3 to 62 per active zone in all the three subpopulations of axon terminals [median (mdn) = 14 and (interquartile range (iqr) = 10–23] determined in 54 active zones of VGAT+, mdn = 18 (iqr = 11–26) in 67 active zones of VGAT- and mdn = 13 (iqr = 9–23) in 90 active zones of VGLUT-1+ neurons from two animals, **Figure 3A**. These quantifications revealed no significant difference between VGAT+ and VGAT- groups of neurons (*p* = 0.22, Mann–Whitney test), neither between VGAT- and VGLUT-1+ terminals (*p* = 0.11; **Figure 3A**). In addition, plotting the number of immunoparticles labeling Ca_v_2.1 against the convex hull area indicated a strong correlation between the number of Ca_v_ channels and the synaptic area in both inhibitory and excitatory boutons [**Figures 3B–D**; Spearman correlation coefficient (r_S_) 0.83, 0.92, and 0.86 for VGAT+, VGAT- and VGLUT-1+ terminals, respectively].

Together, these analyses showed that the number of Ca_v_2.1 channels, despite clear synapse-to-synapse variation, is proportional to the area of active zones suggesting that their overall density in axon terminals of GABAergic and glutamatergic neurons in the stratum radiatum of CA1 is rather constant.

### Ca_v_2.1 Proteins are Organized in Clusters within the Active Zone of Boutons

**FIGURE 4:**
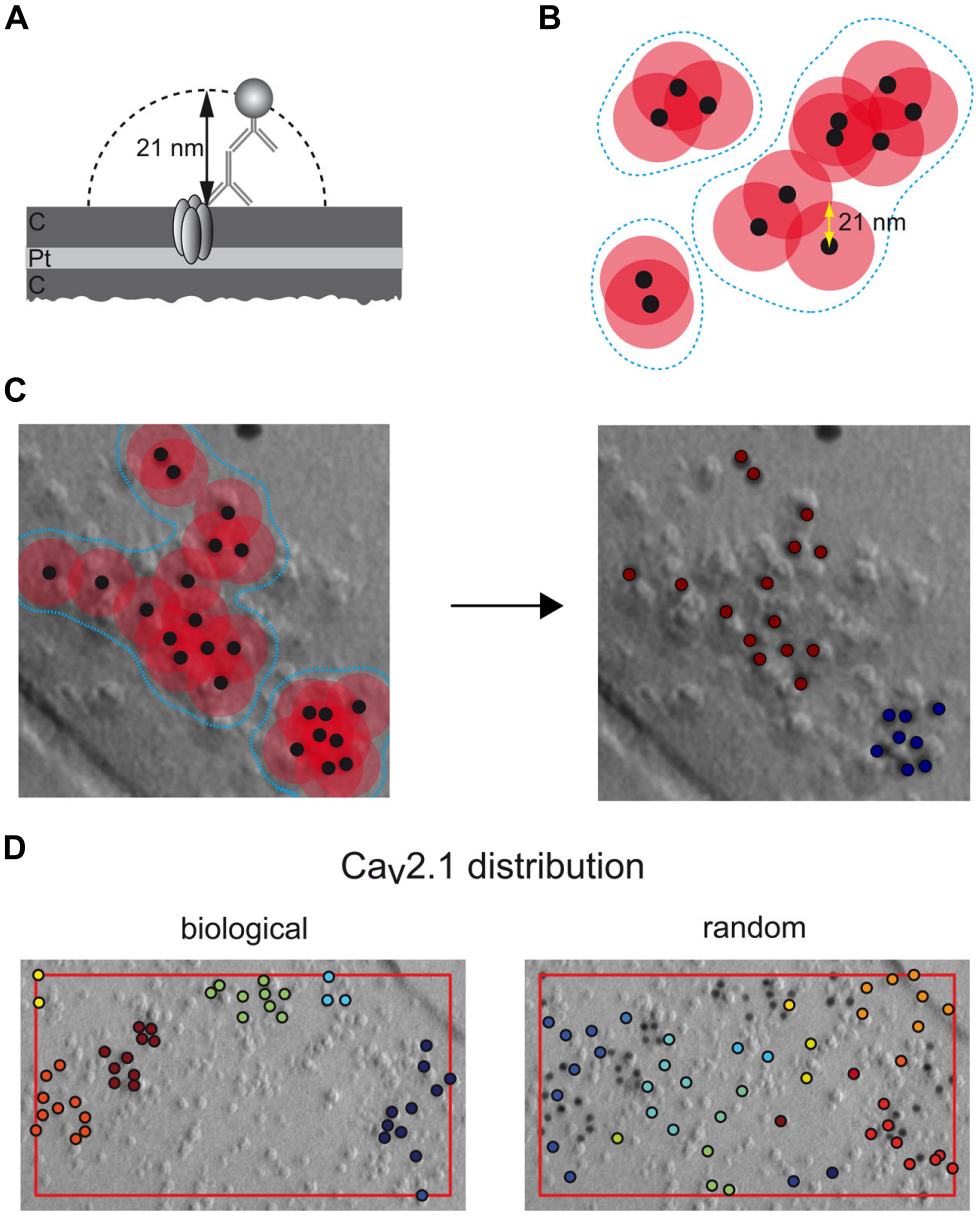
**Rational and operation of the automatized computational procedure used for quantitative assessment of immunoparticle distribution. (A)** Spatial constraints arising from the Ca_v_2.1 (embedded into the carbon (C) and platinum (Pt) layers of the replica) labeling by primary and secondary antibodies (8 nm each) and the gold particle (10 nm). **(B)** Agglomerative clustering of immunoparticles (black dots) using a maximal inter-particle distance of 42 nm (overlapping circles in red); blue broken lines frame individual clusters of immunoparticles derived by this distance constraint (overlapping vs non-overlapping circles). **(C,D)** Operation of the computational procedure: all immunoparticles (black dots) detected in an electron micrograph are evaluated for inter-particle distances based on their 2D-coordinates and grouped into clusters as shown in **(B)**. **(C)** Application to a set of Ca_v_2.1 particles (left image) resulting in the assignment of two distinct clusters (right image). **(D)** Comparison of a clustered distribution (‘biological’) determined by the algorithm for a set of Ca_v_2.1 particles in an axon terminal (area given by box framed in red) and a random sample (‘random’) generated by randomly distributing the same number of particles on an area identical to that determined in the terminal.

For unbiased and quantitative assessment of the distribution of Ca_v_2.1 channels in the active zone, we set up a computational procedure performing automatized distribution analysis based on distances between neighboring immunoparticles (see Materials and Methods). Moreover, the underlying algorithm uses agglomerative clustering of particles when their distances fall below a threshold value that is given by the combined length of the primary and secondary antibodies as well as the radius of the gold particles (21 nm; **Figures 4A,B**). Accordingly, particles located within distances of ≤42 nm from each other are assigned to a common cluster (**Figure 4C**).

**FIGURE 5:**
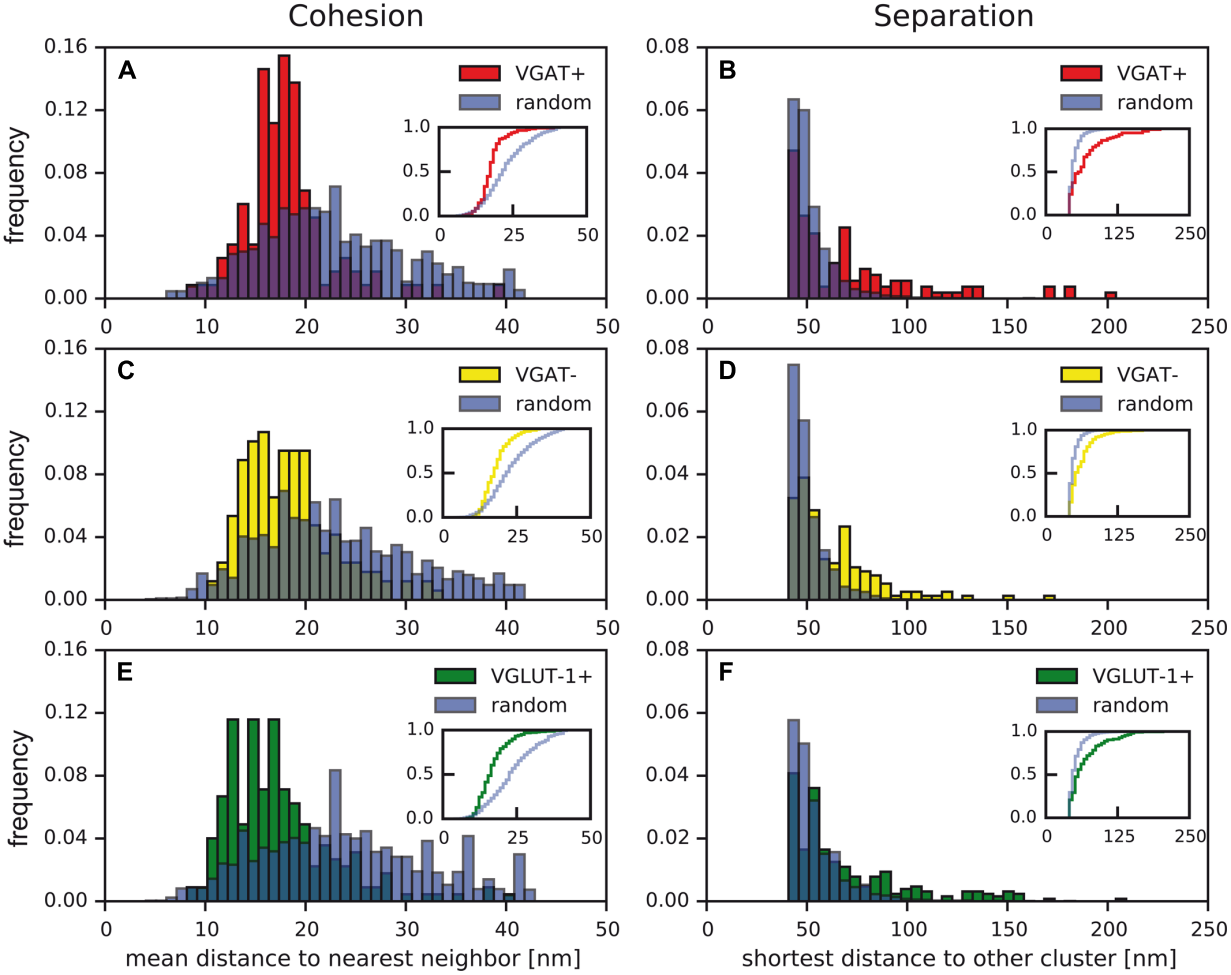
**Distinct distribution of Ca_v_2.1 channels in active zones of presynaptic boutons and in random controls. (A–F)** Distances between nearest Ca_v_2.1 particles (‘cohesion,’ **A,C,E**) and shortest distances between distinct clusters (‘separation,’ **B,D,F**) determined in active zones of VGAT+ (**A,B**; red), VGAT- (**C,D**; yellow), VGLUT-1+ terminals (**E,F**; green) and in random control samples (blue). Insets: cumulative frequency distributions indicating significant differences between biological data (VGAT+, red; VGAT-, yellow; VGLUT-1+, green) and random controls (blue; two-sample Kolmogorov–Smirnov test; *p* < 0.05, exact *p*-values given in **Table 1**).

Using this computer-assisted analysis, we first probed the significance of clustered organization of Ca_v_2.1 channels illustrated above (**Figures 1** and **2**) over random distribution. For this purpose, we determined the distributions of (i) distances between nearest neighboring particles (‘cohesion,’ **Figures 5A,C,E**) and of (ii) shortest distances between two clusters (‘separation,’ **Figures 5B,D,F**), in active zones of VGAT+, VGAT-, and VGLUT-1+ terminals (biological distribution, **Figure 4D**, left panel) and in ‘random controls’ (**Figure 4D**, right panel). For the latter, the same number of particles was positioned randomly within the same area as determined for the respective active zones (red framed box, **Figure 4D**, and see also Materials and Methods).

As illustrated in **Figure 5** and summarized in **Table 1**, the cohesion determined in the various types of boutons was almost identical (values for the median of 18 and 16 nm for VGAT+, VGAT-, and VGLUT-1+ terminals, respectively), but in either case was significantly stronger than the cohesion obtained in random control samples (respective medians of 25, 23, and 23 nm, respectively; *p* < 0.05 two-sample Kolmogorov–Smirnov test, **Figures 5A,C,E**; **Table 1**). Conversely, the separation between clusters was significantly larger in all actual terminals than in random controls (values for the median of 61 and 48 nm (VGAT+), 56 and 48 nm (VGAT-), 58 and 50 nm (VGLUT-1+); *p* < 0.05 two-sample Kolmogorov–Smirnov test, **Figures 5B,D,F**; **Table 1**). These results were independent of the distance constraints, as biological distributions were still significantly different from the respective random controls upon variation of the maximal inter-particle distance between 35 and 55 nm (**Table 2**).

**Table 1:** Analysis of Ca_v_2.1 immunogold distribution in inhibitory and excitatory boutons.

	Biological Median interquartile range (IQR) [nm]	Random Median (IQR) [nm]	*p*-values
**Cohesion**			
VGAT+/random	18 (16–20)	25 (17–27)	5.80E-15
VGAT-/random	18 (15–20)	23 (18–28)	5.70E-20
VGLUT-1+/random	16 (13–20)	23 (18–29)	8.20E-35
VGAT+/VGAT-			0.27
VGAT+/VGLUT-1+			0.00028

	**Biological Median interquartile range (IQR) [nm]**	**Random Median (IQR) [nm]**	***p*-values**

**Separation**			
VGAT+/random	61 (46–81)	48 (45–55)	5.10E-12
VGAT-/random	56 (48–71)	48 (45–54)	2.30E-14
VGLUT-1+/random	58 (48–82)	50 (45–59)	2.80E-18
VGAT+/VGAT-			0.22
VGAT+/VGLUT-1+			0.3

	**Number of clusters Median (IQR) [particles]**	**Particles/cluster Median (IQR) [particles]**	**Cluster diameter Median (IQR) [nm]**

**Cluster parameters**			
VGAT+	2 (1–3)	5 (3–9)	63 (40–110)
VGAT-	2 (1–3)	6 (3–11)	70 (39–110)
VGLUT-1+	2 (1–4)	4 (2–8)	66 (39–140)
***p*-values**			
*p* (VGAT+/VGAT-)	1	0.59	0.5
*p* (VGAT+/VGLUT1+)	0.13	0.11	0.35

*The cohesion, distances between nearest neighboring particles, in vesicular GABA transporter (VGAT+), VGAT-, and VGLUT-1+ terminals was significantly stronger than in random control samples, whereas the separation between clusters was significantly larger in all terminals than in random controls. Parameters such as number of clusters, number of particles for Ca_v_2.1 in individual cluster and diameter of clusters indicate a strikingly similar subcellular arrangement of Ca_v_2.1 channels in inhibitory and excitatory terminals.*

**Table 2:** Parameter scan of inter-particle distances.

	VGAT+		VGAT-		VGLUT-1+	
Distance (nm)	Cohesion	Separation	Cohesion	Separation	Cohesion	Separation
35	1.50E-11	1.50E-07	7.80E-14	1.40E-10	4.10E-25	5.70E-21
36	3.80E-12	1.70E-08	1.10E-14	1.30E-10	2.90E-26	3.30E-21
37	9.50E-15	1.60E-08	7.20E-17	4.00E-12	2.70E-31	8.70E-19
38	2.60E-15	8.70E-10	8.50E-17	9.30E-13	4.40E-31	5.80E-20
39	9.10E-15	2.00E-10	4.50E-17	5.50E-14	1.40E-30	3.80E-24
40	4.00E-16	9.10E-12	4.10E-19	6.00E-14	4.00E-32	4.10E-24
41	1.40E-16	7.90E-12	1.60E-19	9.30E-14	3.80E-32	1.40E-23
**42**	**5.80E-15**	**5.10E-12**	**5.70E-20**	**2.30E-14**	**8.20E-35**	**2.80E-18**
43	1.30E-16	3.90E-12	1.10E-20	1.00E-16	9.10E-34	1.60E-20
44	3.70E-16	5.50E-14	1.10E-21	5.90E-16	5.50E-35	1.10E-19
45	4.50E-16	1.70E-16	5.30E-22	3.90E-17	1.50E-34	3.20E-24
46	4.20E-17	1.70E-16	3.90E-22	4.10E-17	1.30E-35	2.60E-19
47	4.30E-17	1.10E-15	3.40E-23	2.40E-17	1.70E-35	1.90E-18
48	6.90E-17	5.80E-16	3.40E-23	3.10E-19	1.80E-35	4.80E-18
49	1.60E-17	8.30E-17	1.40E-23	2.10E-20	6.60E-36	3.10E-17
50	5.90E-18	3.30E-17	3.60E-24	4.40E-20	4.40E-36	2.30E-18
51	1.10E-17	3.70E-16	2.90E-24	1.00E-18	3.30E-36	5.00E-15
52	2.00E-17	2.50E-16	3.10E-24	4.10E-19	6.30E-36	2.00E-13
53	1.30E-16	3.10E-16	1.80E-24	7.70E-20	5.30E-35	3.60E-14
54	3.40E-16	4.30E-16	3.30E-24	8.30E-19	3.00E-34	1.00E-15
55	1.40E-15	4.80E-15	8.20E-24	6.60E-21	9.60E-33	7.90E-15

*Biological distributions were significantly different from the respective random controls as regards cohesion and separation upon variation of the maximal inter-particle distance between 35 and 55 nm.*

Together, these computational analyses indicated that Ca_v_2.1 channels are in fact organized in clusters over the active zones of both inhibitory and excitatory axon terminals.

### The Nano-Architecture of Ca_v_2.1 Channels is Shared between the Active Zones of Inhibitory and Excitatory Boutons

**FIGURE 6:**
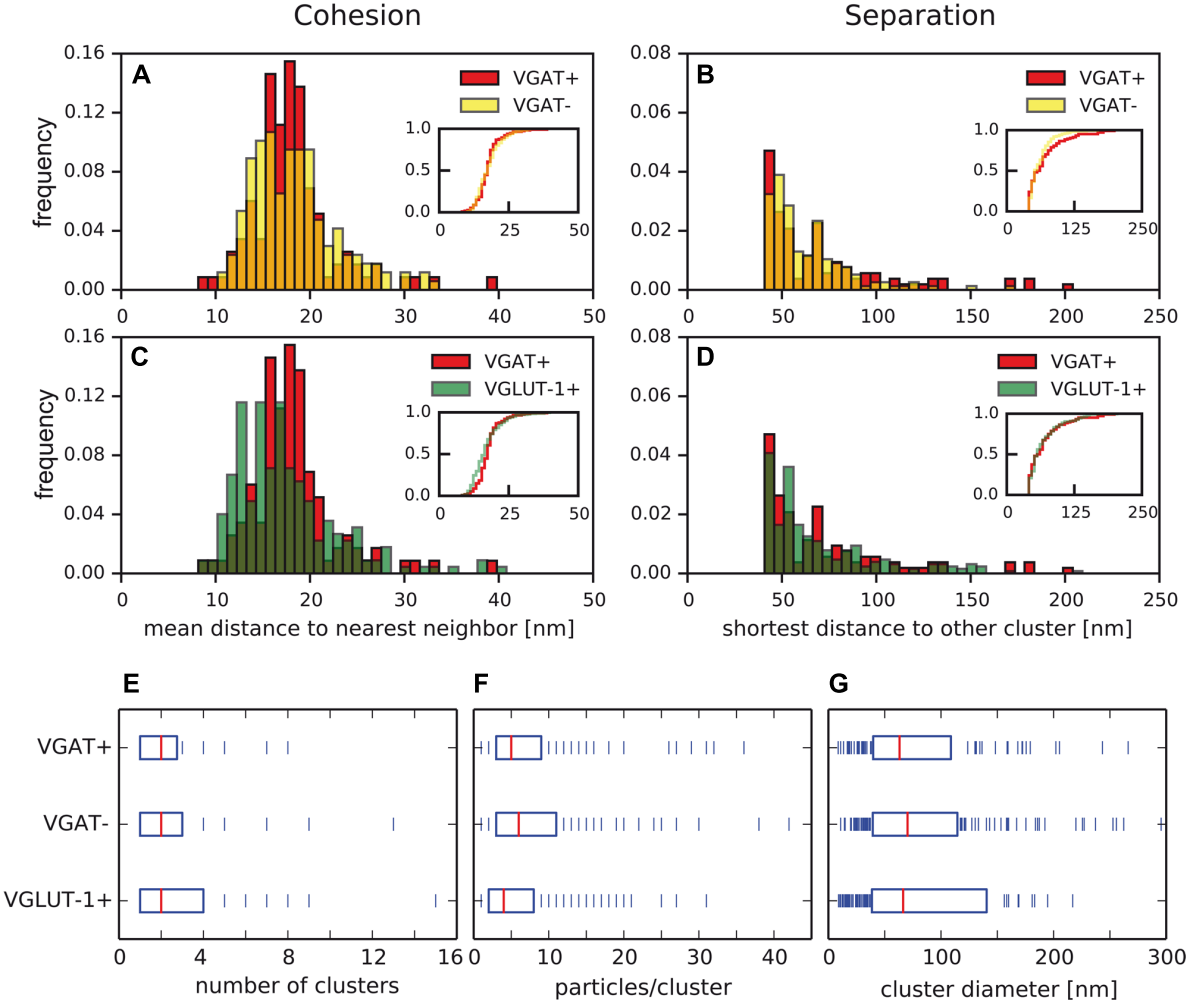
**Properties of Ca_v_2.1 clusters are similar in inhibitory and excitatory axon terminals. (A–D)** Cohesion and separation of Ca_v_2.1 clusters as determined in VGAT+ (red), VGAT- (yellow), and VGLUT-1+ (green) terminals. No significant differences were detected in **(A,B,D),** (*p* < 0.05, two-sample Kolmogorov–Smirnov test), while cohesion in **(C)** was significantly different. **(E–G)** Number of clusters per terminal **(E)**, number of Ca_v_2.1 particles per cluster **(F)**, and cluster diameter **(G)** are determined in the boutons. Boxes indicate median (red) and iqr of the data (blue bars). No significant differences were detected between the data sets from distinct types of boutons (two-sample Kolmogorov–Smirnov test, exact *p*-values given in **Table 1**).

Direct comparison of cohesion and separation of the Ca_v_2.1 clustering did not reveal statistically significant differences between inhibitory and excitatory terminals (**Figures 6A–D**), strongly suggesting a more general architecture that is shared among presynaptic compartments of different types of neurons. The slight difference observed for the cohesion between VGAT+ and VGLUT-1+ terminals is most likely due to the distinct primary antibodies that target different epitopes on the channel protein and may distinctly impact the spatial arrangements of the gold grains and, therefore, the distances between nearest neighboring particles.

In subsequent analyses, the computational procedure was, therefore, used for a more detailed investigation of the parameters characterizing the architecture of the Ca_v_2.1 clusters in inhibitory and excitatory boutons. At this end, we determined (i) the number of clusters in presynaptic terminals, (ii) the number of immunogold particles for Ca_v_2.1 forming an individual cluster as well as, (iii) the diameter of the area covered by a cluster as accessible to our SDS-FRL configuration. The number of clusters varied roughly between 1 and 10 and averaged to two clusters per terminal in both inhibitory and excitatory boutons (**Figure 6E**; **Table 1**). Similarly, the number of Ca_v_2.1 immunogold particles integrated in the same cluster varies over a wide range (3–40) averaging to a value of 5 in all the three types of presynaptic terminals (**Figure 6F**; **Table 1**). Finally, the diameter of the individual clusters ranged from 10 to 250 nm and exhibit mean values of 63, 70, and 66 nm in VGAT+, VGAT-, and VGLUT-1+ terminals, respectively (**Figure 6G**; **Table 1**).

Together, these quantitative data unequivocally indicate a strikingly similar subcellular arrangement of presynaptic Ca_v_2.1 channels in inhibitory and excitatory axon terminals in the stratum radiatum of hippocampal CA1 region.

## Discussion

In the present study we investigated and compared the ultrastructural organization of Ca_v_2.1 channels in axon terminals of inhibitory (VGAT+) and excitatory (VGAT- and VGLUT-1+) neurons in the stratum radiatum of the CA1 hippocampal area using high-resolution SDS-FRL electron microscopy. Furthermore, we used an automatized computational analysis to compare the precise spatial arrangement of Ca_v_2.1 channels in the two subpopulations of axon terminals and suggest a common nano-architecture of the P/Q-type Ca^2+^ channel.

Immunoelectron microscopy unequivocally revealed enrichment of Ca_v_2.1 channels in the active zone of boutons as well as a close spatial relationship of the channel subunit to the presynaptic proteins RIM1/2, established components of the Ca_v_2 channel networks and major regulators of the coupling between Ca^2+^ channels and Ca^2+^ sensors of exocytosis (Müller et al., 2010; Han et al., 2011; Kaeser et al., 2011; Gundelfinger and Fejtova, 2012; Südhof, 2013). Quantitative morphological as well as detailed computational analysis further demonstrated a high degree of structural similarity between inhibitory and excitatory terminals with respect to clustering and average number of Ca_v_2.1 channels in the presynaptic membrane. These findings are consistent with the clustered distribution and estimates of the number of Ca_v_2.1 channels in the active zone of calyx of Held (Nakamura et al., 2015), cerebellar parallel fibers (Indriati et al., 2013; Schmidt et al., 2013; Baur et al., 2015), and hippocampal CA3 principal cells synapsing on either other CA3 (Holderith et al., 2012) or CA1 pyramidal (Ermolyuk et al., 2013) neurons. Interestingly, the size of the clusters and the number of Ca_v_2.1 proteins per cluster (4–6) correlate well with estimates derived from electrophysiological studies (Bucurenciu et al., 2010) and biochemical/proteomic analysis (Müller et al., 2010) that found a small number of Ca_v_ channels located within a distance of less than 100 nm from the release machinery at central synapses. Therefore, our data provide qualitative and quantitative proof of principle that GABAergic and glutamatergic synapses in the stratum radiatum of the CA1 area share a common nano-architecture of Ca_v_2.1 channels making extensive use of tight coupling between the Ca_v_2.1 channels and Ca^2+^ sensors for fast transmitter release.

In that respect, the Ca_v_2.1 clusters reflect the molecular basis for local Ca^2+^ signaling in ‘Ca^2+^ nano-domains’ (Neher, 1998; Fakler and Adelman, 2008). The tight coupling of Ca_v_2.1 channels and Ca^2+^ sensors of exocytosis ensures high reliability in vesicle release (Scimemi and Diamond, 2012), reduced synaptic delay and duration of the release period as well as increased ratio of synchronous and ‘entopic’ (active zone) release resulting in similar high temporal precision of both GABAergic and glutamatergic transmission (Bucurenciu et al., 2008, 2010; Eggermann et al., 2012; Nadkarni et al., 2012).

In summary, our results demonstrate a large morphological homogeneity in the two non-overlapping populations of synapses (**Figure 2A**) suggesting that the processes and mechanisms underlying the formation of the Ca_v_2.1 nano-architecture and the evoked release of neurotransmitters are similar between inhibitory and excitatory central synapses (Xu et al., 2009; Eggermann et al., 2012). Regarding that the protein nano-environment of Ca_v_2 channels is highly complex (Berkefeld et al., 2006; Müller et al., 2010) consisting of quite a variety of auxiliary proteins and regulators (Arikkath and Campbell, 2003; Dolphin, 2012) that together form the channel-associated networks regulating the local Ca^2+^ signaling (Evans and Zamponi, 2006; Han et al., 2011; Hoppa et al., 2012; Davydova et al., 2014), it is conceivable that the assembly and operation of Ca_v_2.1 channel clusters can be dynamically regulated. In this respect, further extensive quantitative research is required to identify and localize additional components of the Ca_v_2.1 channel-associated networks and to unravel the synapse- and/or state-specific properties of the nano-environments of P/Q-type Ca^2+^ channels as well as their concerted (Wheeler et al., 1994; Spafford and Zamponi, 2003; Williams et al., 2012) implication in the homeostatic control of cortical synapses function.

## Author Contributions

DA, BF, and AK designed the project; DA and DB performed computational cluster analysis; DA and AK performed SDS-FRL immunoelectron microscopy; MW provided reagents; NS provided knock-out tissues; DA, DB, BF, and AK analyzed data; DA, BF, and AK wrote the paper.

## Conflict of Interest Statement

The authors declare that the research was conducted in the absence of any commercial or financial relationships that could be construed as a potential conflict of interest.
